# Prognosis value of biomarkers in severe sepsis and septic shock

**DOI:** 10.1186/cc14147

**Published:** 2015-03-16

**Authors:** MV De La Torre-Prados, A García-de la Torre, R Escobar-Conesa, J Perez-Vacas, A Puerto-Morlán, E Cámara-Sola, A García-Alcántara

**Affiliations:** 1 Hospital Virgen de la Victoria, Málaga, Spain; 2 Puerto Real University Hospital, Puerto Real, Cádiz, Spain

## Introduction

The purpose of the study was to assess the prognosis value of pro-adrenomedullin (pADM), C-reactive protein (CRP) and procalcitonin (PCT), lactate (LT), albumin (ALB), cholesterol (CHOL), white blood cell (WBC) and severity score in patients with severe sepsis or septic shock.

## Methods

A prospective, observational study in adult patients with severe sepsis or septic shock in a polyvalent ICU. Demographics, severity scores (APACHE II and SOFA) and all of the biomarkers were studied within 24 hours from septic shock onset. Descriptive and comparative statistical analysis was performed using the statistical software packages SPSS v.15 and MedCalc^® ^9.2.1.0.

## Results

We analyzed 246 consecutive episodes of severe sepsis (38%) or septic shock (62%). The 28-day mortality was 36.2%. The profile of dead patients had a significantly higher average age (65 (IQR: 75.5 to 57.5) vs. 63 (47 to 72); *P *< 0.06), APACHE II (27 (22 to 30) vs. 23 (18 to 27); *P *< 0.001) and SOFA (11 (9 to 12.75) vs. 9 (7 to 10); *P *< 0.001). CRP (168.4 (106 to 285) vs. 165.4 (87.8 to 275) mg/dl; *P *= NS), PCT (6.5 (0.94 to 23.8) vs. 5.8 (0.97 to 19.59) ng/ml; *P *= NS) and WBC 14.7 (9.5 to 21.4) vs. 12.9 (5.5 to 17.5); *P *= NS) were increased in those who died, but CHOL (102 (75 to 134) vs. 108 (86 to 141) mg/dl; *P *= NS) had lower values. These differences were significant in pADM (2.46 (1.21 to 4.89) vs. 1.68 (0.94 to 3.32) nmol/l; *P *= 0.012), LT (2.6 (1.6 to 3.94) vs. 1.6 (1.2 to 2.43) mmol/l; *P *< 0.001) and ALB (2 (1.55 to 2.38) vs. 2.22 (1.96 to 2.7) g/dl; *P *= 0.001).

## Conclusion

The protein pADM, LT and ALB showed good prognosis accuracy when measured on admission of septic patients to the ICU.

